# ﻿Four new species of the genus *Hendelia* Czerny, 1903 (Diptera, Clusiidae) from China

**DOI:** 10.3897/zookeys.1212.127558

**Published:** 2024-09-19

**Authors:** Shuai-Lai Yang, Xin-Ming Yin, Yu-Qiang Xi

**Affiliations:** 1 Department of Entomology, Henan Agricultural University, No. 95 Wenhua Road, Jinshui District, Zhengzhou 450003, Henan Province, China Henan Agricultural University Zhengzhou China

**Keywords:** China, Clusiodinae, druid flies, identification key, new species, taxonomy

## Abstract

Four new species of the genus *Hendelia* Czerny, 1903 collected from China, are described as new to science: *H.latustigenis***sp. nov.**, *H.macrocera***sp. nov.**, *H.deltoides***sp. nov.** and *H.flavida***sp. nov.** An updated key to the species of *Hendelia* from China is presented.

## ﻿Introduction

Clusiidae (Diptera, Acalyptratae) are species commonly called “druid flies”, and comprise 14 genera and at least 640 species (Lonsdale 2017; Yang et al. 2021). The genus *Hendelia* Czerny, 1903 belongs to the subfamily Clusiodinae. Its generic characteristics include small lobate surstyli that are directed towards each other, a male perianal region (desclerotized triangle surrounding the anus) that is longer than wide, an ejaculatory apodeme that is widest apically, and a plumose arista (modified in some species) (Lonsdale and Marshall 2007). Features shared predominantly with its sister genus *Clusiodes* include strong interfrontal setae, reduced anterior and posterior fronto-orbitals, and highly derived genitalia including a keel-like phallapodeme and a subapical sclerotized disc on the ventral receptacle of the female (Lonsdale and Marshall 2011).

There are 59 described species of *Hendelia* distributed worldwide; 29 species are distributed in the Palaearctic and Oriental regions; four species are known in Japan: *H.plumosa* (Sasakawa), *H.angulosa* (Sueyoshi), *H.plumipes* (Sasakawa) and *H.beckeri* Czerny; three species are known in Russia (Far East): *H.quatuorsetosa* (Mamaev), *H.bisetosa* (Mamaev) and *H.unica* (Mamaev) (Sasakawa 1964; Mamaev 1974; Sasakawa 1987; Lonsdale 2017). Three species are known in China: *Hendeliabeckeri* Czerny,1903, *H.formosana* (Hennig,1938) and *H.freyi* (Soós,1963); these species were found in Sichuan, Taiwan and Zhejiang provinces (Hennig 1938; Soós 1963; Lonsdale and Marshall 2007; Lonsdale 2017).

In this study, four new species are described from China: *Hendelialatustigenis* sp. nov., *H.macrocera* sp. nov., *H.deltoides* sp. nov. and *H.flavida* sp. nov. A key to the known species of *Hendelia* from China and some Palaearctic and Oriental species is presented.

## ﻿Material and methods

Genitalia preparations were made by removing and macerating the apical portion of the abdomen in glacial acetic acid, then rinsing in distilled water before storage in glycerin-filled microvials. Specimens were examined and photographed using a Leica M205A microscope. After examination, genitalia were transferred to fresh glycerin and stored in a microvial on the pin below the specimen or moved to an ethanol tube together with the wet specimens. The coordinates of the collection site were obtained by asking the collectors. Image plates were post-processed with Adobe PHOTOSHOP CC 2019 Extended.

Specimens examined were deposited in the Entomological Museum of Henan Agricultural University (HAU), Zhengzhou. Terminology follows Lonsdale and Marshall (2006). The M_1_ ratio is defined as the length of the ultimate section of wing vein M divided by the length of the penultimate section (Lonsdale and Marshall 2006).

## ﻿Taxonomy

### ﻿Key to species of *Hendelia* of China and nearby countries

**Table:** 

1	Two fronto-orbital setae	**2**
–	Three fronto-orbital setae	**7**
2	Cerci large, height is 3.5 times the width	***H.beckeri* Czerny**
–	Cerci small	**3**
3	Mesontum dark brown without stripes	**4**
–	Mesontum with brown stripes	**5**
4	Vibrissae strongly elongated and thickened, setae on the surface of surstylus dense	***H.quatuorsetosa* (Mamaev)**
–	Vibrissae normal, setae on the surface of surstylus sparse	***H.bisetosa* (Mamaev)**
5	Setae completely black, middle part of hypandrium with a finger-like projection	***H.latustigenis* sp. nov.**
–	Setae with brown, hypandrium without finger-like projection	**6**
6	Legs yellow; postocellar seta smaller than ocellar seta	***H.angulosa* (Sueyoshi)**
–	Legs yellow except fore tarsus and most of fore tibia brownish-black, and hind tibia with pale brown rings near both apices; postocellar seta and ocellar seta almost the same length	***H.plumosa* (Sasakawa)**
7	Vibrissae long and strong; interfrontal setae also very strong and nearly parallel	***H.macrocera* sp. nov.**
–	Vibrissae and interfrontal setae normal, no obvious extension	**8**
8	Face entirely black	***H.unica* (Mamaev)**
–	Face not entirely black	**9**
9	Setae yellow (at least for the head); mesonotum without stripes	**10**
–	Setae black; mesontum with stripes	**11**
10	Mesonotum yellow, anterior margin and postpronotum brown	***H.formosana* (Hennig)**
–	Mesonotum black, mesonotum pollinose, weakly shining	***H.plumipes* (Sasakawa) (female)**
11	Gena with 3 or 4 subgenal setae; thorax with 1 wide brownish-black longitudinal stripe	***H.freyi* (Soós)**
–	Gena not 3 subgenal setae; thorax with 3 thin pale brown stripes	**12**
12	Front half of the face dark brown and the back half yellow; abdomen mainly dark brown	***H.deltoides* sp. nov.**
–	Frons and face light yellow; abdomen mainly light yellow	***H.flavida* sp. nov.**

#### ﻿ Hendelia deltoides sp. nov.

Taxon classificationAnimaliaDipteraClusiidae

3C9271A9-7C71-523A-95F9-2772F636ADF1

https://zoobank.org/17AF0495-DCC1-4FEF-AFB5-5094581FA3A6

Figs 1–6 , 31

##### Type material.

***Holotype***: • 1 ♂; China, Shaanxi, Zhouzhi, Taibai Mt.; 33°57'18″N, 107°45'48″E; 1711 m; 2015. VII. 29; leg. Ning Jinjin. ***Paratypes***: • 1 ♂; same data as for holotype; 2015.VII.30; leg. Li Xuankun; • 1 ♂; China, Chongqing, Yingtiaoling Nature Reserve; 31°28'44.93"N, 109°53'26.19"E; 1373 m; 2022. VI. 26; leg. Xu Rongzhen; • 1 ♂; China, Chongqing, Yingtiaoling Nature Reserve; 31°28'44.93"N, 109°53'26.19"E; 1373 m; 2022.VI.26; leg. GuanYuliang.

##### Diagnosis.

Head mostly yellow; palpus white; face dark brown and back half yellow. Thorax mainly yellow; with 3 light brown stripes extending to the scutellum. Surstylus nearly triangular, 1/5 length of genitalia, apex without spines. Hypandrium with 4 setae.

**Figures 1–6.**
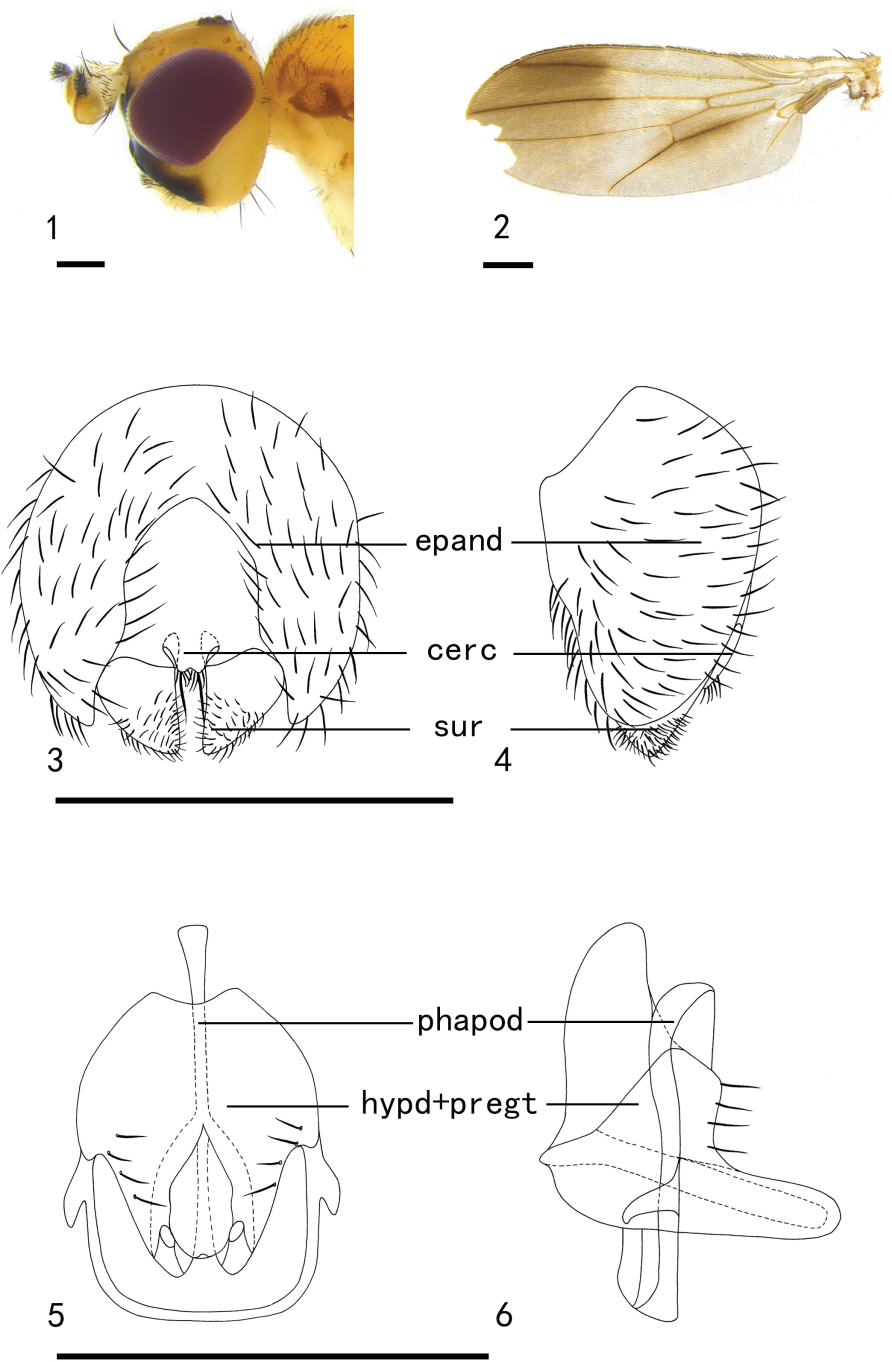
*Hendeliadeltoides* sp. nov. (male) **1** head, lateral view **2** wing **3** epandrium, cerci, and surstyli, posterior view **4** epandrium, cerci, and surstyli, lateral view **5** hypandrial complex, posterior view **6** hypandrial complex, lateral view. Scale bars: 0.1 mm. Abbreviations: epand – epandrium; cerc – cerci; sur – surstylus; hypd – hypandrium; pregt – pregonite

##### Description.

**Male.** Body length 4.4–4.6 mm; Wing length 4.2–4.4 mm.

***Head*** (Fig. 1) width 1.49 mm, mostly yellow; palpus white; face dark brown and the back half yellow; frons yellow, 3 fronto-orbital setae, 1 interfrontal seta; occiput yellow; gena yellowish, more than 1/4 height of eye; antenna yellow, pedicel length 0.20 mm, arista very short plumose; 1 ocellar seta and 1 postocellar seta.

***Thorax*** (Fig. 31) width 1.04 mm, mainly yellow; scutum with 3 light brown stripes extending to the scutellum; scutellum yellow, anepisternum yellow with brown spots, katepisternum yellowish; prescutellar seta absent; 1 postpronotal seta, 2 notopleural setae, 1 postsutural supra-alar seta, 1+2 dorsocentral setae, dark brown; intra-alar seta absent, 2 postalar setae, dark brown, the posterior seta tiny; 1 lateral scutellar seta, 1 apical scutellar seta, strong and dark brown; anepisteral seta absent, 1 katepisternal seta. Legs light yellow. Wing (Fig. 2) with light brown spots on distal 1/3 and on M_4_; the M_1_ ratio 4.9; halter white.

***Abdomen*** mainly brown; anterior edge of each abdominal segment yellowish, setae and setulae dark brown. Male genitalia (Figs 3–6): epandrium height and width almost equal. Cerci 0.9 times as high as wide, with small setae on outer surface and one long seta at apex. Surstylus nearly triangular, 1/5 length of genitalia, apex without spines. Hypandrium with 4 setae; pregonite slightly chitinized; postgonite with small setae at apex; side of phallus slightly chitinized, distiphallus long, membranous, transparent.

**Female.** Unknown.

##### Distribution.

China (Shaanxi, Chongqing).

##### Etymology.

The specific name “*deltoides*” refers to the surstylus, which is nearly triangular.

##### Remarks.

The species is similar to *H.angulosa* (Sueyoshi), but it can be separated by the face black and the three fronto-orbital setae. In *H.angulosa* (Sueyoshi), the face is yellow and the head has only two fronto-orbital setae (Sueyoshi 2006).

#### ﻿ Hendelia macrocera sp. nov.

Taxon classificationAnimaliaDipteraClusiidae

1C5D8FB7-F7CF-5333-AC80-3A32B6B98C2D

https://zoobank.org/20740AED-CE48-476B-8C41-0550DE4E2482

Figs 7–12 , 32

##### Type material.

***Holotype***: • ♂; China, Shaanxi, Zhouzhi, Houzhenzi; 33°51'3.056″N, 107°50'37.924″E; 1545 m; 2015. VIII. 2; Leg. Li Xuankun. ***Paratypes***: • 2 ♂♂; same data as holotype.

##### Diagnosis.

Head mostly yellowish, including face and frons; palpus yellowish-white; interfrontal seta long and strong; pedicel very long. Scutum with Y-shaped brown stripe extending to scutellum. Surstylus nearly “triangular” in shape. Hypandrium with two pairs of ventromedial setae.

##### Description.

**Male.** Body length 4.3–4.5 mm, Wing length 4.2–4.4 mm.

**Figures 7–12.**
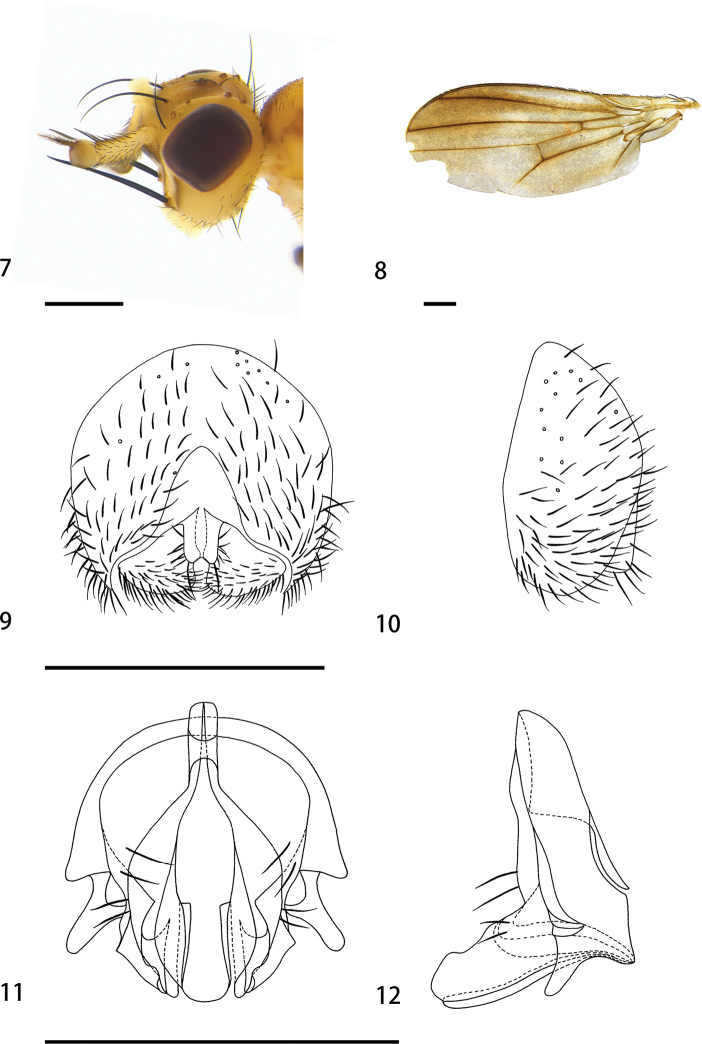
*Hendeliamacrocera* sp. nov. (male) **7** head, lateral view **8** wing **9** epandrium, cerci, and surstyli, posterior view **10** epandrium, cerci, and surstyli, lateral view **11** hypandrial complex, posterior view **12** hypandrial complex, lateral view. Scale bars: 0.1 mm.

***Head*** (Fig. 7) width 1.63 mm, mostly yellowish; palpus yellowish-white; 3 fronto-orbitals, setae small; interfrontal seta long and strong, some small setulae around the interfrontal seta; occiput yellowish with sparse small setae; gena more than 1/3 height of eye; antenna yellow, pedicel length 0.15 mm, twice length of first flagellomere, scape brown, arista sparsely short plumose; 1 ocellar seta and 1 postocellar seta, small; vibrissae very strong.

***Thorax*** (Fig. 32) width 1.26 mm, mainly yellowish; scutum with brown Y-shaped stripe extending onto scutellum; scutellum yellowish on both sides, anepisternum with pale brown longitudinal stripes, katepisternum yellowish. Prescutellar seta absent; 1 postpronotal seta, 2 notopleural setae, 1 postsutural supra-alar seta, 1+2 dorsocentral setae, dark brown; intra-alar seta absent, 2 postalar setae, dark brown, posterior seta tiny; 2 lateral scutellar setae, 1 apical scutellar seta, strong and dark brown; 1 anepisternal seta, 1 katepisternal seta. Leg yellowish. Wing (Fig. 8) with light brown spots on distal 1/3, along veins R_2+3_, the M_1_ ratio 2.8; halter white.

***Abdomen*** brown, setae and setulae dark brown. Male genitalia (Figs 9–12): epandrium 0.8 times as high as wide. Cerci small, the height and width almost equal, slightly separated distally, with small setae on the outer surface and one longer seta at apex. Surstylus nearly triangular in shape, 0.4 times the length of the genitalia, apex without spines. Hypandrium with 2 long setae at the apex. Distiphallus long, membranous.

**Female.** Unknown.

##### Distribution.

China (Shaanxi).

##### Etymology.

The specific name “*macrocera*” refers to the long antennae.

##### Remarks.

The new species is similar to *H.extensicornis* Frey, but can be separated by the long, strong vibrissae; the interfrontal setae are also very strong and nearly parallel. In *H.extensicornis* Frey, the vibrissae are small and the interfrontal setae are small and crossed (Frey 1960).

#### ﻿ Hendelia latustigenis sp. nov.

Taxon classificationAnimaliaDipteraClusiidae

E844E26D-742B-5374-8B59-9F702937D911

https://zoobank.org/3300FE52-4C28-498A-B722-411FFB7DBBC8

Figs 13–18

##### Type material.

***Holotype***: • 1 ♂; China, Shaanxi, Zhouzhi, Taibai Mt.; 1711 m; 33°57'18″N, 107°45'48″E; 2015. VII. 30; leg. Li Xuankun. ***Paratypes***: • 1 ♂; same date as holotype.

##### Diagnosis.

Head mostly yellowish, palpus yellowish-white. Mesontum with 2 brown stripes extending to the anterior margin of scutellum; hypandrium with medial finger-like projection that has 2 long setae apically.

##### Description.

**Male.** Body length 4.2–4.5 mm, Wing length 4.1–4.3 mm.

**Figures 13–18.**
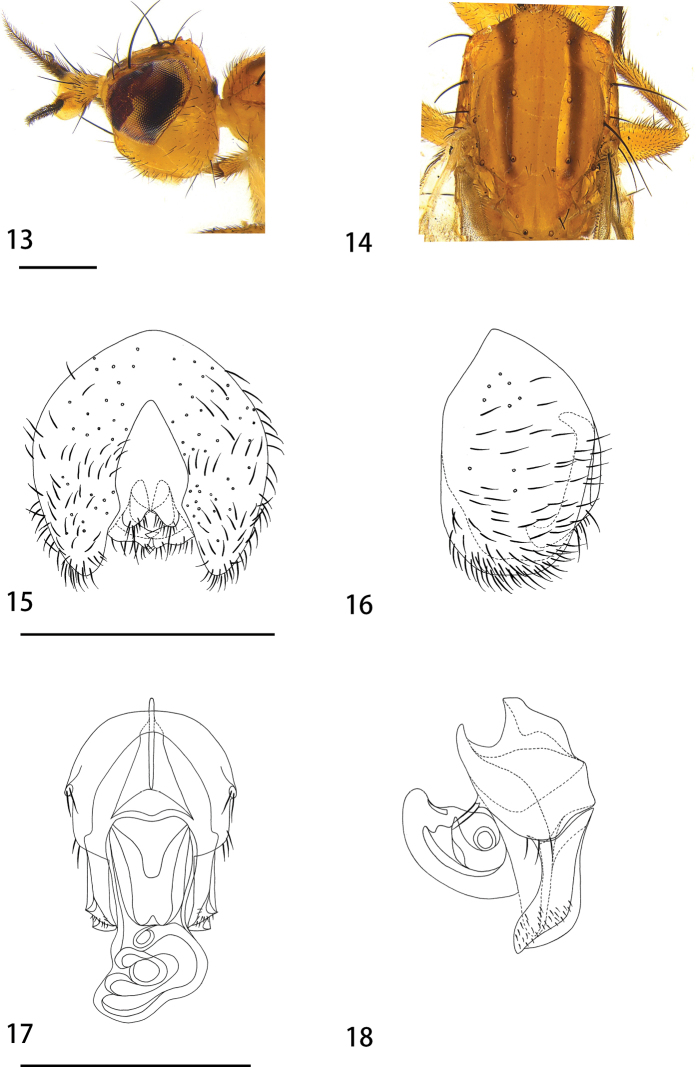
*Hendelialatustigenis* sp. nov. (male) **13** head, lateral view **14** mesothorax, dorsal view **15** epandrium, cerci, and surstyli, posterior view **16** epandrium, cerci, and surstyli, lateral view **17** hypandrial complex, posterior view **18** hypandrial complex, lateral view. Scale bars: 0.1 mm.

***Head*** (Fig. 13) width 1.28 mm, mostly yellowish; palpus yellowish-white; face and frons yellowish; 2 fronto-orbitals, the posterior seta 4 times longer than anterior seta; the interfrontal seta same length as anterior fronto-orbital; occiput yellowish, with small seta on lower edge; gena yellowish, more than 1/6 eye height; antenna yellow, pedicel length 0.16 mm, arista sparsely short plumose; 1 ocellar seta and 1 postocellar seta.

***Thorax*** width 1.22 mm, mainly light yellow; with narrow brown stripe outside dorsocentral row extending to the anterior margin of scutellum (Fig. 14); scutellum, anepisternum and katepisternum yellowish; prescutellar seta absent; 1 postpronotal seta, 2 notopleural, 1 postsutural supra-alar seta, 1+2 dorsocentral setae, dark brown; intra-alar seta absent, 2 postalar setae, brown, with posterior seta tiny; 2 lateral scutellar setae, 1 apical scutellar seta, strong and dark brown; 1 anepisternum seta, 1 katepisternal seta. Leg light yellow; halter white.

***Abdomen*** brown; setae and setulae dark brown. Male genitalia (Figs 15–18): epandrium 1.2 times higher than wide. Cercus height 1.8 times width, separated on distal 1/2, with small setae on outer surface and one long seta at apex. Surstylus 1/4 length of genitalia, apex without spines. hypandrium with medial finger-like projection that has 2 long setae apically, posterior part of hypandrium with 2 or 3 medium setae; pregonite slightly chitinized, with small apical setae.

**Female.** Unknown.

##### Distribution.

China (Shannxi).

##### Etymology.

The specific name “*latustigenis*” refers to the relatively wide gena.

##### Remarks.

The new species is distinct from other species in the middle part of the hypandrium with a finger-like projection at the tip of which there are two long setae, and in the lower part of the hypandrium with two or three medium setae. The wings of the type specimen are damaged.

#### ﻿ Hendelia flavida sp. nov.

Taxon classificationAnimaliaDipteraClusiidae

D12EB5CD-D391-5B65-817A-66BE1388C1BF

https://zoobank.org/60030C35-B4CD-417F-AC8F-22574FECDEA9

Figs 19–24 , 34

##### Type material.

***Holotype***: • ♂; China, Yunnan, Gongshan, Xianjiudan; 27°56'13.524″N, 98°19'55.250″E; 1679 m; 2013. VII. 3; leg. Li Xuankun. ***Paratypes***: • 2 ♂♂; same data as holotype.

##### Diagnosis.

Body mostly yellow. Head and thorax yellowish; palpus white; scutum with 3 indistinct brown stripes. Cerci very small, outline nearly that of an equilateral triangle, fully fused. Pregonite with 4 medial setae, lacking any other smaller setae or setulae.

##### Description.

**Male.** Body length 3.4–3.6 mm, Wing length 2.2–2.4 mm

**Figures 19–24.**
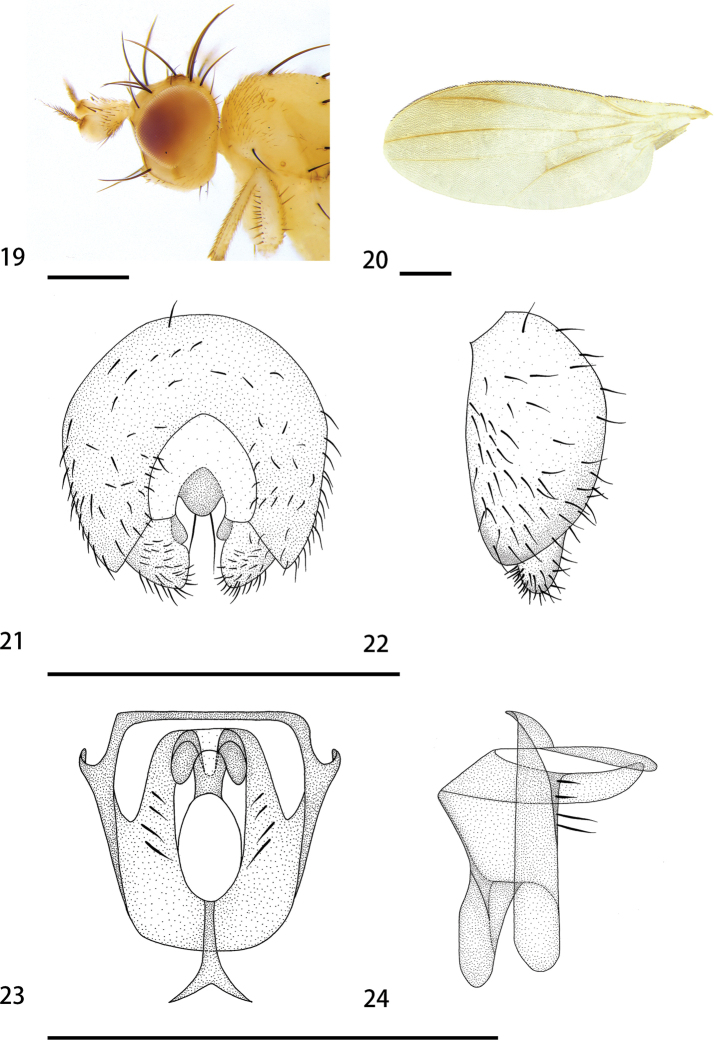
*Hendeliaflavida* sp. nov. (male) **19** head, lateral view **20** wing **21** epandrium, cerci, and surstyli, posterior view **22** epandrium, cerci, and surstyli, lateral view **23** hypandrial complex, posterior view **24** hypandrial complex, lateral view. Scale bars: 0.1 mm.

***Head*** (Fig. 19) width 0.50 mm, yellowish; palpus white; face yellowish; frons yellowish, 3 fronto-orbital setae, first seta and last seta small, medium seta strong, 1 interfrontal seta, strong; occiput yellowish with small sparse brown setae; gena yellowish, more than 1/5 height of eye; antenna yellowish, pedicel length 0.19 mm, first flagellomere brown apically, arista sparse short plumose; 1 strong ocellar seta, equal in length to interfrontal seta, 1 small postocellar seta, almost as long as anterior fronto-orbital seta.

***Thorax*** width 0.98 mm, mainly yellowish with 3 indistinct brown stripes; scutellum, anepisternum and katepisternum yellowish; prescutellar seta absent; 1 postpronotal seta, 2 notopleural setae, 1 postsutural supra-alar seta, 1+2 dorsocentral setae, dark brown, the last long; intra-alar seta absent, 2 postalar setae, dark brown; 1 lateral scutellar seta, 1 apical scutellar seta, strong; 1 anepisternum seta, 1 katepisternal seta. Legs light yellow with tibiae brown. Wing (Fig. 20) clouded on distal 2/5, becoming darker anteriorly, and around middle of M_1_; M_1_ ratio 6; halter white.

***Abdomen*** dark yellow and white; setae and setulae brown. Male genitalia (Figs 21–24): epandrium height and width subequal in length. Cerci very small, outline almost that of equilateral triangle, not separated, height 0.9 times width. Surstylus 0.4 times length of epandrium, with many small setae on inner and outer surfaces, apex without spines. Pregonite with 4 medial setae, lacking any other smaller setae or setulae. Distiphallus short, membranous.

**Female.** Unknown.

##### Distribution.

China (Yunnan).

##### Etymology.

The specific name “*flavida*” refers to the colour of this species, which is mostly pale yellow.

##### Remarks.

The new species is similar to *H.plumosa* (Sasakawa), but it differs in having a yellowish head, indistinct stripes on the scutum, and fully fused cerci. In *H.plumosa* (Sasakawa), the head is yellow, the thorax is more distinctly striped, and the cerci are separated (Sueyoshi 2006).

#### ﻿ Hendelia beckeri

Taxon classificationAnimaliaDipteraClusiidae

Czerny, 1903

C6ADF6E9-DC9E-5F0A-B700-E6CF32E02E5F

Figs 25–30 , 33

##### Material examined.

•1 ♂; China, Hebei, Xinlong, Wulingshan, Shibatan; 40°33'34.524″N, 117°28'53.187″E; 2019. VI. 12; leg. Yang Ding; • 2 ♂♂; China, Sichuan, Pingwu, Wanglang National Natural Reserve; 32°54'29"N, 104°09'28"E; 2480 m; 2017. VIII. 1; leg. Xi Yuqiang; • 1 ♂; China, Hebei, Xinglong, Wulingshan, Dujuanfeng; 40°31'49.569″N, 117°30'26.503″E; 2019. VI. 11; leg. Yang Ding.

##### Distribution.

China (Sichuan, Zhejiang, Hebei); central Europe to Norway and Finland; eastern and northern coasts of Black Sea; central and eastern Russia, Japan, South Korea.

**Figures 25–30.**
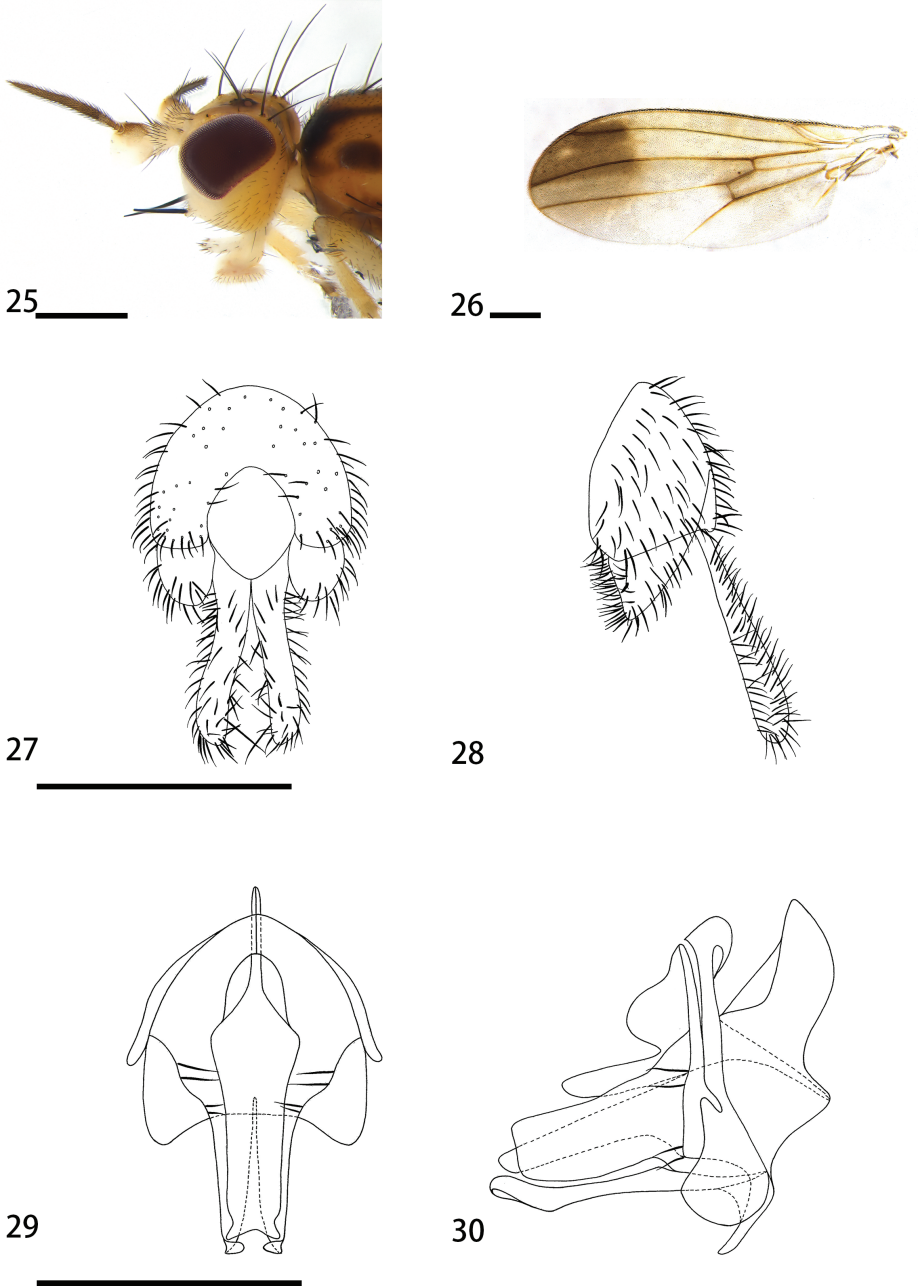
*Hendeliabeckeri* (male) **25** head, lateral view **26** wing **27** epandrium, cerci, and surstyli, posterior view **28** epandrium, cerci, and surstyli, lateral view **29** hypandrial complex, posterior view **30** hypandrial complex, lateral view. Scale bars: 0.1 mm.

**Figures 31–34.**
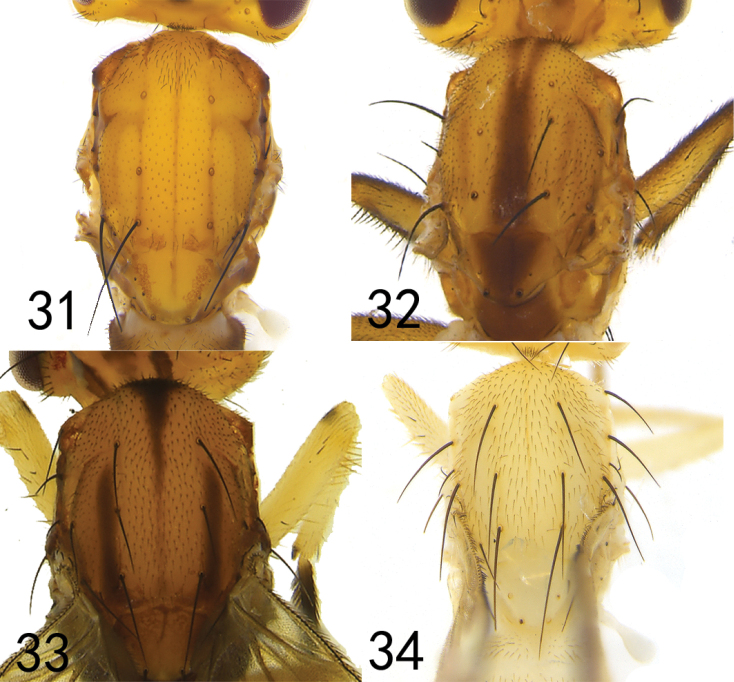
Mesothorax, dorsal view **31***Hendeliadeltoides* sp. nov. **32***Hendeliamacrocera* sp. nov. **33***Hendeliabeckeri* (male) **34***Hendeliaflavida* sp. nov.

## Supplementary Material

XML Treatment for Hendelia deltoides

XML Treatment for Hendelia macrocera

XML Treatment for Hendelia latustigenis

XML Treatment for Hendelia flavida

XML Treatment for Hendelia beckeri

## ﻿Additional information

### Author ORCIDs

Shuai-Lai Yang https://orcid.org/0009-0002-6813-8949

Xin-Ming Yin https://orcid.org/0000-0003-3936-3557

Yu-Qiang Xi https://orcid.org/0000-0002-3285-8904

### Data availability

All of the data that support the findings of this study are available in the main text or Supplementary Information.
